# Dipyridamole potentiates the in vitro activity of MTA (LY231514) by inhibition of thymidine transport

**DOI:** 10.1054/bjoc.1999.1020

**Published:** 2000-02

**Authors:** P G Smith, E Marshman, D R Newell, N J Curtin

**Affiliations:** Cancer Research Unit, University of Newcastle upon Tyne, Medical School, Framlington Place, Newcastle upon Tyne, NE2 4HH, UK

**Keywords:** dipyridamole, LY231514, thymidine transport, hypoxanthine transport

## Abstract

The novel pyrrolopyrimidine-based antifolate LY231514 (MTA), inhibits multiple folate-requiring enzymes including thymidylate synthase, glycinamide ribonucleotide formyltransferase and dihydrofolate reductase. Both thymidine and hypoxanthine are required to reverse MTA growth inhibition in leukaemia and colon cancer cells. Prevention of MTA growth inhibition by thymidine and/or hypoxanthine was investigated in two human lung (A549, COR L23) and two breast (MCF7, T47D) tumour cell lines, and the effect of the nucleoside/base transport inhibitor dipyridamole (DP) on thymidine and hypoxanthine rescue defined. MTA IC_50_values (continuous exposure three population doublings) were: A549–640 n M, COR L23–28 n M, MCF7–52 n M and T47D–46 n M. Thymidine (1 μM) completely prevented growth inhibition at the MTA IC_50_in all cell lines. At 10 × IC_50_, growth inhibition was only partially reversed by thymidine (≤ 10 μM); both thymidine and hypoxanthine (30 μM) being required for complete reversal, reflecting the multi-targeted nature of MTA. Growth inhibition by MTA was not affected by hypoxanthine alone. A non-toxic concentration (1 μM) of DP prevented thymidine/hypoxanthine rescue of MTA indicating that DP may potentiate MTA activity by preventing nucleoside and/or base salvage. Thymidine transport was inhibited by ≥ 89% by 1 μM DP in all cell lines, whereas hypoxanthine transport was inhibited only in A549 and MCF7 cells. Therefore, prevention of end-product reversal of MTA-induced growth inhibition by DP can be explained by inhibition of thymidine transport alone. © 2000 Cancer Research Campaign


*British Journal of Cancer* (2000) **82**(4), 924–930
© 2000 Cancer Research Campaign
DOI: 10.1054/ bjoc.1999.1020, available online at http://www.idealibrary.com on IDEAL®

### Dipyridamole potentiates the in vitro activity of MTA (LY231514) by inhibition of thymidine transport

**PG Smith, E Marshman, DR Newell and NJ Curtin**

Cancer Research Unit, University of Newcastle upon Tyne, Medical School, Framlington Place, Newcastle upon Tyne, NE2 4HH, UK

**Summary** The novel pyrrolopyrimidine-based antifolate LY231514 (MTA), inhibits multiple folate-requiring enzymes including thymidylate synthase, glycinamide ribonucleotide formyltransferase and dihydrofolate reductase. Both thymidine and hypoxanthine are required to reverse MTA growth inhibition in leukaemia and colon cancer cells. Prevention of MTA growth inhibition by thymidine and/or hypoxanthine was investigated in two human lung (A549, COR L23) and two breast (MCF7, T47D) tumour cell lines, and the effect of the nucleoside/base transport inhibitor dipyridamole (DP) on thymidine and hypoxanthine rescue defined. MTA $IC_{50}$ values (continuous exposure three population doublings) were: A549–640 nM, COR L23–28 nM, MCF7–52 nM and T47D–46 nM. Thymidine (1 μM) completely prevented growth inhibition at the MTA $IC_{50}$ in all cell lines. At 10 × $IC_{50}$, growth inhibition was only partially reversed by thymidine (≤ 10 μM); both thymidine and hypoxanthine (30 μM) being required for complete reversal, reflecting the multi-targeted nature of MTA. Growth inhibition by MTA was not affected by hypoxanthine alone. A non-toxic concentration (1 μM) of DP prevented thymidine/hypoxanthine rescue of MTA indicating that DP may potentiate MTA activity by preventing nucleoside and/or base salvage. Thymidine transport was inhibited by ≥ 89% by 1 μM DP in all cell lines, whereas hypoxanthine transport was inhibited only in A549 and MCF7 cells. Therefore, prevention of end-product reversal of MTA-induced growth inhibition by DP can be explained by inhibition of thymidine transport alone. © 2000 Cancer Research Campaign

**Keywords:** dipyridamole; LY231514; thymidine transport; hypoxanthine transport

The novel pyrrolopyrimidine-based antifolate MTA (LY231514, *N*-[4[2-(2-amino-3,4-dihydro-4-oxo-7H-pyrrolo[2,3-d]pyrimindin-5-yl)-ethyl]-benzoyl]-L-glutamic acid) inhibits several folate-dependent enzymes involved in de novo nucleotide biosynthesis, including thymidylate synthase (TS: E.C.2.1.1.45), dihydrofolate reductase (DHFR: E.C.1.5.1.3) and glycinamide ribonucleotide formyltransferase (GARFT: E.C.2.1.2.2) (Shih et al, 1997). In addition, MTA is one of the best known substrates for mammalian folylpolyglutamate synthase (E.C.6.3.2.17) and polyglutamation increases the intracellular retention of MTA and MTA-induced inhibition of TS and GARFT. MTA is a potent inhibitor of tumour cell growth in vitro, e.g. in CCRF-CEM leukaemia cells ($IC_{50}$ = 25 nM), GC3/C1 colon cells ($IC_{50}$ = 34 nM) and HCT-8 illoecaecal cells ($IC_{50}$ = 220 nM) (Shih et al, 1997). The multi-targeted nature of MTA has been confirmed in whole cell investigations where end-product reversal studies have shown that both thymidine and hypoxanthine are required to completely reverse the antiproliferative effect of MTA (Shih et al, 1997). MTA is currently undergoing phase II clinical trials in a range of tumour types where promising activity has been observed (Calvert and Walling, 1998).

Resistance to antifolates may be mediated by a number of intrinsic or acquired mechanisms in cancer cells. One intrinsic mechanism is circumvention of the block of de novo nucleotide synthesis via the salvage of extracellular preformed nucleosides and nucleobases (Weber, 1983). For example, extracellular thymidine can be phosphorylated by thymidine kinase (E.C.2.7.1.21) to dTMP hence by-passing inhibition of TS. Similarly, hypoxanthine can be converted to IMP by hypoxanthine-guanine phosphoribosyl transferase (E.C.2.4.2.8) thereby overcoming inhibition of de novo purine synthesis. A number of factors may contribute to the overall activity of the salvage pathway in tumours. In particular, the activities of salvage enzymes in cancer cells are higher than those of de novo synthesis (Weber, 1983) and the activity of the salvage pathway increases in parallel with malignancy (Kinsella and Haran, 1991). Furthermore, the breakdown of dead and dying cells in vivo may increase the concentration of salvageable nucleosides and bases in the vicinity of the tumour. Thus nucleoside and nucleobase salvage may compromise the in vivo activity of antimetabolites, and inhibition of the salvage pathway should therefore overcome this form of resistance.

The transport of salvageable nucleosides and nucleobases across the cell membrane is the first step in the salvage pathway. Nucleoside transport is mediated largely by equilibrative transporters at physiological nucleoside concentrations (Plagemann and Wohlhueter, 1984*b*). The cardiovascular agent dipyridamole (DP) inhibits nucleoside transport in all human cell lines tested, and has been used successfully to potentiate the activity of antifolates in vitro. For example, DP potentiates CB3717 (an antifolate TS inhibitor) by preventing the salvage of thymidine (Curtin and Harris, 1988). Similarly, the cytotoxicity of methotrexate, which inhibits both de novo thymidylate and purine biosynthesis, is increased by DP in a variety of human and animal cells through inhibition of thymidine and/or hypoxanthine uptake (Nelson and Drake, 1984; Van Mouwerik et al, 1987; Hughes and Tattersall, 1989; Chan and Howell, 1990). DP has been shown to inhibit hypoxanthine transport in some cell lines but not others (Plagemann and Wohlhueter, 1984*a*), and has been used to

*Received 24 May 1999*
*Revised 14 September 1999*
*Accepted 22 September 1999*

*Correspondence to:* NJ Curtin produce a cell line-specific blockade of hypoxanthine rescue from antipurine antifolate-induced growth inhibition (Turner et al, 1997; Marshman et al, 1998).

The aim of the investigation reported here was to determine the effect of DP on reversal of MTA-induced growth inhibition by thymidine and hypoxanthine in cells previously characterized for their sensitivity to DP-mediated inhibition of hypoxanthine rescue from antipurine antifolate toxicity (Marshman et al, 1998). Four cell lines representing common human malignancies, two non-small-cell lung carcinoma (NSCLC: A549 and COR L23) and two breast carcinoma (MCF7 and T47D) were selected. In A549 and MCF7 cells DP prevents hypoxanthine rescue from antipurine antifolate-induced growth inhibition, whilst in COR L23 and T47D cells DP has no effect on hypoxanthine rescue (Marshman et al, 1998). On the basis of these data the cell lines were described as having either *d*ipyridamole *s*ensitive transport (*ds*, A549 and MCF7) or *d*ipyridamole-*i*nsensitive hypoxanthine transport (*di*, COR L23 and T47D). The mechanism of rescue from MTA-induced growth inhibition was examined by investigation of thymidine and hypoxanthine uptake and its inhibition by DP in the four cell lines.

### MATERIALS AND METHODS

### Materials

LY231514 (MTA, *N*-[4[2-(2-amino-3,4-dihydro-4-oxo-7H-pyrrolo[2,3-d]pyrimindin-5-yl)-ethyl]-benzoyl]-L-glutamic acid) was a gift from Eli Lilly and Co., USA. MTA was dissolved in $dH_2O$ and stored at 4°C. Thymidine, hypoxanthine and dipyridamole (Sigma Chemical Company, Poole, UK) were dissolved in $dH_2O$, 0.1 M sodium hydroxide (NaOH) and 100% dimelthyl sulphoxide (DMSO) respectively, and stored in the dark at 4°C for a maximum of 4 weeks. All other reagents were obtained from Sigma unless otherwise stated.

### Cell culture

COR L23 (a kind gift from Dr P Twentyman, MRC Clinical Oncology and Radiotherapeutics Unit, Cambridge, UK) and A549 (National Cancer Institute, National Institutes of Health, Bethesda, MD, USA) non-small-cell lung carcinoma; MCF7 and T47D breast adenocarcinoma (European Collection of Animal Cell Cultures) were adapted to growth in RPMI-1640 medium supplemented with 1000 units $ml^{-1}$ penicillin, 100 µg $ml^{-1}$ streptomycin (Gibco, Paisley, UK) and 10% (v/v) fetal calf serum that had been dialysed (four changes of 9 volumes phosphate-buffered saline (PBS) + 1 g $L^{-1}$ activated charcoal) to remove salvageable nucleosides and bases. All cells were maintained as exponentially growing cultures. Cells were tested every month for mycloplasma contamination using a Hoechst 33258 DNA fluorescence-based technique (Chen, 1977) and found to be negative.

### Growth inhibition assays

The optimum cell seeding density was determined to be $1 \times 10^3$ cells $well^{-1}$ for A549 and COR L23 cells, $2 \times 10^3$ for T47D cells and $3 \times 10^3$ for MCF7 cells. Cells were incubated in 96-well plates (Nunc) and grown at 37°C in a humidified atmosphere at 5% carbon dioxide. Following incubation the plates were washed with PBS, fixed with methanol:acetic acid (3:1 v/v), washed, air-dried and stained with sulphorhodamine B (SRB) as described previously (Skehan et al, 1990). The optical density relative to an air blank was measured on a Dynatech MR7000 96-well microtitre plate reader (Dynatech) using a 570 nm filter.

Cells in logarithmic phase growth were harvested with trypsin, seeded at $1–3 \times 10^3$ in 100-µl in 96-well plates and allowed to attach to the plate overnight. The medium was replaced with that containing semi-logarithmic dilutions of MTA for A549, CORL 23 and MCF7 cells and logarithmic dilutions of MTA for T47D cells for a period equivalent to three population doubling times (72, 96, 144 and 96 h for A549, COR L23, MCF7 and T47D cells respectively). The $IC_{50}$ values (concentration causing 50% inhibition of growth) were determined by non-linear regression fitting of a sigmoid curve to the data using GraphPad PRISM™ (San Diego, CA, USA) software. End product reversal experiments and investigations of the effect of DP were conducted using a fixed concentration of MTA at the $IC_{50}$ or $10 \times IC_{50}$ determined as above for each cell line. Thus A549, COR L23, MCF7 and T47D cells were exposed to 700 nM or 7000 nM, 20 nM or 200 nM, 50 nM or 500 nM and 50 nM or 500 nM MTA respectively, ± 1–10 µM thymidine, 1–30 µM hypoxanthine and/or 1 µM DP in 1% (v/v) DMSO for three cell doublings followed by SRB assay as described above.

### Thymidine transport assays

A modified rapid mixing technique (Wolhueter et al, 1978), combined with the inhibitor-stop method of Paterson et al (1981), was used as described below. Uptake was measured over a 12-s period to calculate initial rates of transport as opposed to subsequent metabolism. For each thymidine concentration, six replicate 0.4 ml microtest tubes (BDH) were prepared by adding 50 µl 3 M potassium hydroxide (KOH) followed by a layer of Dow Corning silicone oil (specific gravity of 1.028). Cells in logarithmic phase growth were harvested with PBS containing 400 mg $L^{-1}$ EDTA and centrifuged at 1850 ***g*** at 4°C for 5 min. The cell pellet was re-suspended in transport buffer (130 mM sodium chloride (NaCl), 5 mM potassium chloride (KCl), 1 mM magnesium chloride ($MgCl_2$) 5 mM $NaH_2PO_4$, 10 mM glucose, 25 mM HEPES at pH 7.1) and centrifuged again before re-suspension in transport buffer at $4 \times 10^7$ cells $ml^{-1}$. Cell suspensions (333 µl) were incubated with 50 µl DMSO ± DP (20 × final concentration) 283 µl transport buffer and incubated at 21°C for 6 min. One to $2 \times 10^6$ cells were then layered over the oil layer in six separate microfuge tubes. Cell number and viability were confirmed by counting and trypan blue exclusion. Transport was initiated by addition of radiolabel mixture (containing $^3H$-thymidine (final specific activity 6.2 Gbq $mmol^{-1}$), $^{14}C$-sucrose (final specific activity 7.4 Gbq $mmol^{-1}$) and transport buffer resulting in a final concentration of 100 µM thymidine) at 1-s time intervals. After exposure to thymidine for 2, 4, 6, 8, 10 and 12 s transport was terminated by the addition of 400 µM DP. The tubes were immediately transferred to an Eppendorf (5415 C) microcentrifuge (Eppendorf, Hamburg, Germany) and pulse centrifuged at 6700 ***g*** to pellet the cells into the KOH layer. The tubes were left for 1 h to allow the cells to dissolve in the KOH layer and the tubes were then cut through the oil layer and the bottom section containing the KOH transferred to a scintillation vial. The KOH was neutralized by the addition of 1 ml of 0.25 M acetic acid and 10 ml Optiphase Hisafe (Wallac, Milton Keynes, UK) scintillation fluid was added. The samples were then counted

© 2000 Cancer Research Campaign on a Wallac 1410 liquid scintillation counter (Pharmacia Wallac, Milton Keynes, UK). Since $^{14}$C-sucrose does not enter the cells, the ratio of $^{14}$C:$^{3}$H was used to calculate the amount of contaminating $^{3}$H-thymidine in the extracellular space in each sample. The contaminating $^{3}$H-thymidine was subtracted from the total $^{3}$H-thymidine count in order to calculate the amount of thymidine transport at each time point.

### Hypoxanthine transport assay

Cells in logarithmic phase growth were harvested with PBS containing 400 mg L$^{-1}$ EDTA and centrifuged at 1850 ***g*** at 4°C for 5 min. The cell pellet was re-suspended in hypoxanthine transport buffer (140 mM NaCl, 10 mM HEPES at pH 7.4) and centrifuged again before resuspending in transport buffer. Cells were incubated with 5% (v/v) DMSO (control) or dipyridamole dissolved in DMSO for 5 min at 21°C. A 100 µl aliquot of this suspension was pipetted into 0.4-ml microfuge tubes containing a layer of 200 µl Dow Corning silicone oil (final specific gravity 1.028) over 50 µl 3 M KOH. Transport was initiated by the addition of 50 µl of radiolabel containing [8-$^{3}$H]-hypoxanthine (final specific activity 0.13 Gbq mmol$^{-1}$) and [U-$^{14}$C]-sucrose (final specific activity 7.4 Gbq mmol$^{-1}$) giving a final concentration of 30 µM HPX in transport buffer. Transport was measured over ten seconds and was stopped by centrifugation at 6700 ***g*** for 2 min of the cells through the oil layer into the KOH layer. Tubes were then processed as described for thymidine transport measurements.

### RESULTS

The cell doubling time and optimum seeding density to give exponential growth during the exposure period was determined for each cell line by daily SRB assays of replicate 96-well plates seeded with varying initial cell densities. The doubling times estimated in this manner are given in Table 1. Cell growth inhibition was determined following continuous exposure to increasing concentrations of MTA for at least three population doublings. The $IC_{50}$ concentrations, defined as the concentration of drug required to inhibit cell growth by 50%, are given in Table 1. A broad range of sensitivities to MTA was observed with A549 cells ($IC_{50}$ = 640 nM) being the least sensitive and COR L23 cells ($IC_{50}$ = 28 nM) being the most sensitive (Table 1).

End product reversal studies in CCRF-CEM cells have shown that thymidine can prevent MTA-induced growth inhibition at concentrations at or below the MTA $IC_{50}$, but that thymidine and hypoxanthine are required for complete rescue at concentrations above the MTA $IC_{50}$ (Shih et al, 1997). To investigate if a similar pattern of end product reversal was observed in breast and lung cancer cell lines, each cell line was exposed to the $IC_{50}$ and 10 × $IC_{50}$ concentration of MTA in the presence of increasing concentrations of thymidine (1 µM, 5 µM and 10 µM) (Figure 1). All cell lines displayed complete reversal of MTA-induced growth inhibition at the $IC_{50}$ concentration in the presence of thymidine. In A549 and COR L23 cells, 1 µM thymidine was sufficient to afford complete reversal of MTA-induced growth inhibition at the $IC_{50}$ concentration; however, in MCF7 and T47D cells 5 µM thymidine was required. In all cells, only partial reversal of MTA-induced growth inhibition was observed following treatment with MTA at a concentration of 10 × the $IC_{50}$, even when the concentration of thymidine was raised to 10 µM. Thymidine alone (1–10 µM) did not affect the growth of any of the cell lines (data not shown).

Hypoxanthine alone (1–30 µM) conferred no reversal of growth inhibition following exposure to 10 × $IC_{50}$ concentrations of MTA in any of the four cell lines tested (Figure 2), and 1 µM hypoxanthine did not enhance the partial rescue afforded by thymidine in any cell line. However, the combination of 30 µM hypoxanthine + 1 µM thymidine gave complete reversal of the growth inhibitory effects of MTA at a concentration of 10 × the $IC_{50}$.

In cells exposed to 10 × $IC_{50}$ concentrations of MTA, DP (1 µM) inhibited the partial rescue afforded by thymidine in all four cell lines (Figure 3). DP has previously been shown to inhibit hypoxanthine rescue from antipurine antifolates in A549 and MCF7 cells but not in COR L23 and T47D cells (Marshman et al, 1998). However, in the study demonstrated here, DP completely inhibited the reversal of MTA-induced growth inhibition by the combination of thymidine and hypoxanthine in all cells tested. Interestingly, A549 cell growth was reduced to 11 ± 4% of control by the combination of MTA (at 10 × $IC_{50}$) + 1 µM DP and, in the additional presence of thymidine and hypoxanthine reduced to 10 ± 3% of control, this was more growth inhibitory than the cell growth produced by MTA (at 10 × $IC_{50}$) alone which was reduced to 19 ± 2% of control.

To confirm that the inhibition of rescue produced by DP was due to the inhibition of thymidine and hypoxanthine uptake, transport assays were performed using 100 µM $^{3}$H-thymidine and 30 µM $^{3}$H-hypoxanthine in the presence or absence of 1 µM and 10 µM DP. One µM DP was sufficient to inhibit thymidine transport by > 89% in all four cell lines (Table 2). Hypoxanthine transport was significantly reduced in A549 cells by 45% ($P < 0.05$) and by 60% ($P < 0.05$) in MCF7 cells at 1 µM DP and was inhibited further by 69% and 89% in A549 and MCF7 cells, respectively, at 10 µM DP. In contrast, DP (10 µM) did not cause a significant inhibition of hypoxanthine transport in COR L23 cells (25%, $P > 0.1$) cells or in T47D cells (30%, $P > 0.1$).

**Table 1** MTA-induced inhibition of cell growth

| Cell line | Tissue type | Doubling time (h) | Exposure period (h) | MTA $IC_{50}$ concentration (nM) |
|---|---|---|---|---|
| A549 | Lung | 20 | 72 | 640±25 |
| COR L23 | Lung | 31 | 96 | 28±14 |
| MCF-7 | Breast | 45 | 144 | 52±5 |
| T47D | Breast | 32 | 96 | 46±1 |

Cell doubling times were determined using log-linear plots of daily SRB assay data using GraphPad PRISM™. Growth inhibition was determined following exposure to MTA for the time periods given. The $IC_{50}$ is the concentration of MTA required to inhibit the growth of the cell population by 50%. Data are the mean ± standard deviation from three independent experiments.

 *© 2000 Cancer Research Campaign*

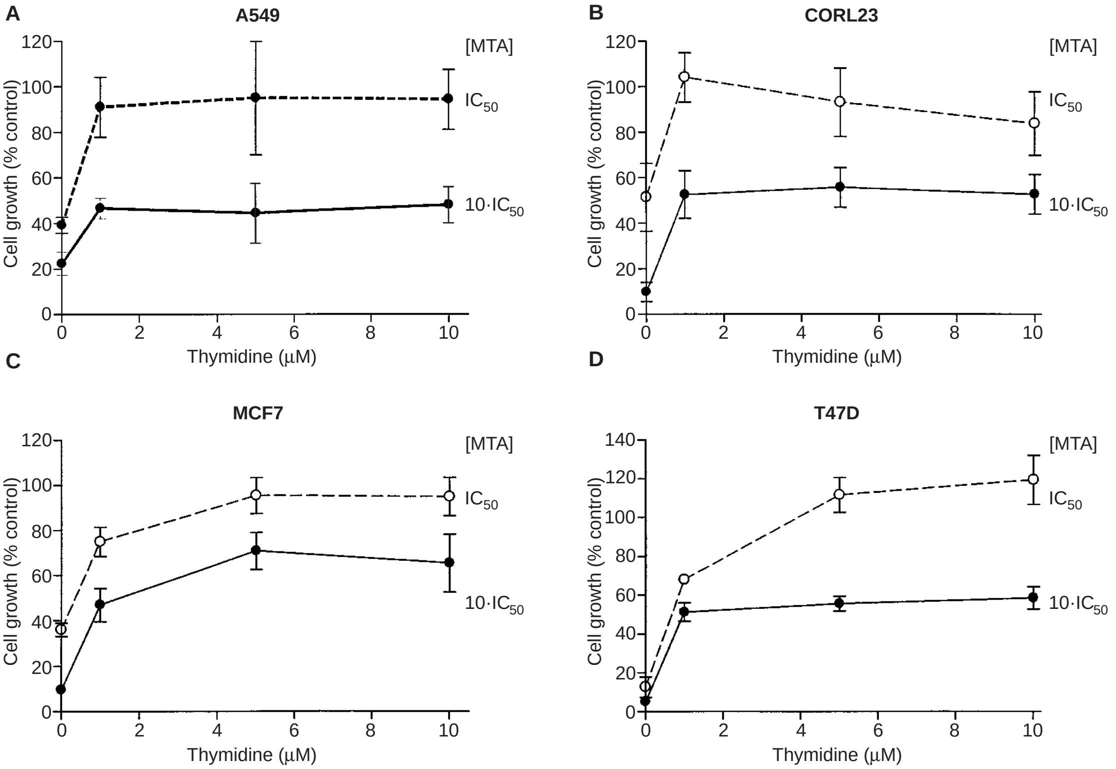

**Figure 1** Thymidine rescue from MTA-induced growth inhibition in (**A**) A549, (**B**) COR L23, (**C**) MCF7 and (**D**) T47D cells. Cell growth was determined by the SRB assay following exposure to an $IC_{50}$ MTA concentration (solid line, closed symbols), or 10 × the $IC_{50}$ concentration (broken line, open symbols), in the presence of increasing thymidine concentrations for three cell doublings. Data are the mean ± standard deviation from three independent experiments

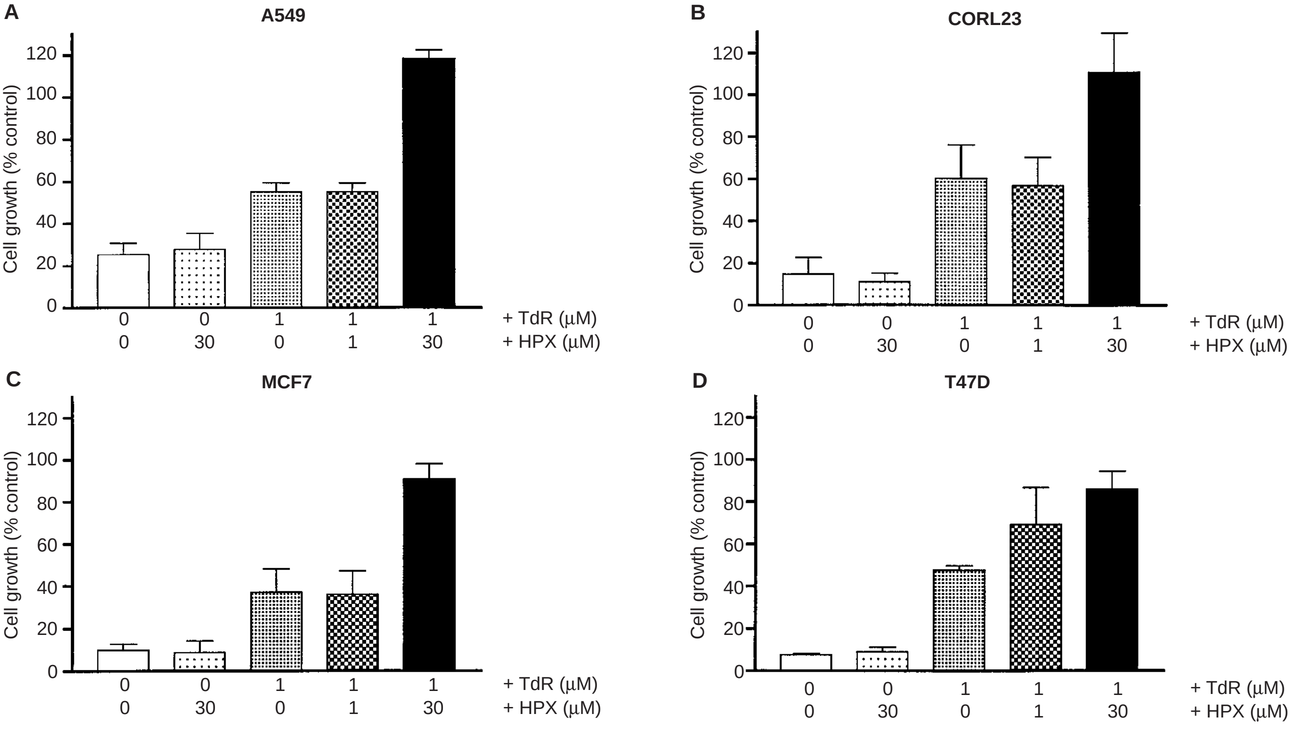

**Figure 2** Thymidine and hypoxanthine rescue from MTA growth inhibition in (**A**) A549, (**B**) COR L23, (**C**) MCF7 and (**D**) T47D cells. Cell growth was determined by the SRB assay following exposure to 10 × the MTA $IC_{50}$ concentration, in the presence or absence of 1 µM thymidine and 1 or 30 µM hypoxanthine for three cell doublings. Data are the mean ± standard deviation from three independent experiments

© 2000 Cancer Research Campaign

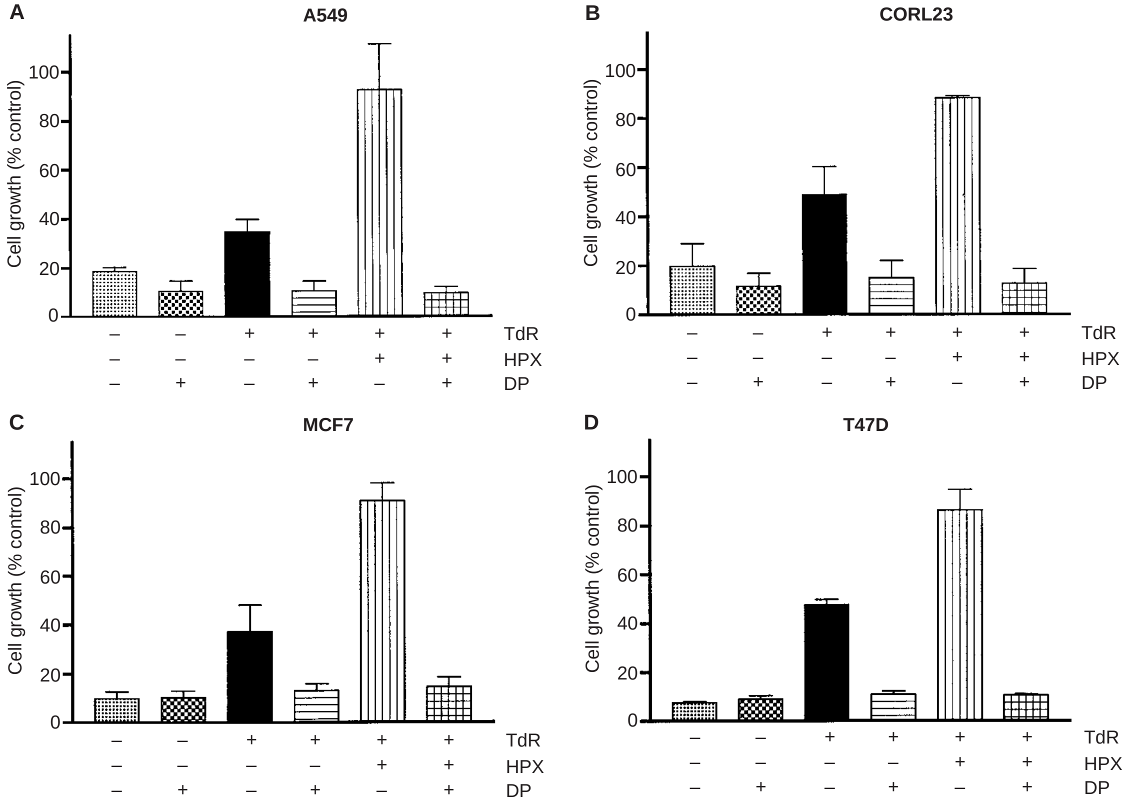

**Figure 3** Prevention of thymidine and hypoxanthine rescue from MTA-induced growth inhibition by dipyridamole in (**A**) A549, (**B**) COR L23, (**C**) MCF7 and (**D**) T47D cells. Cell growth was determined by the SRB assay following exposure to 10 × the MTA $IC_{50}$ concentration in the presence or absence of 1 μM thymidine, 30 μM hypoxanthine and 1 μM dipyridamole for three cell doublings. Data are the mean ± standard deviation from three independent experiments

**Table 2** Inhibition of thymidine (100 μM) and hypoxanthine (30 μM) transport by dipyridamole

| Cell line | Thymidine transport: % inhibition | | Hypoxanthine transport: %inhibition | |
|---|---|---|---|---|
| | + 1 μM DP | + 10 μM DP | + 1 μM DP | + 10 μM DP |
| A549 | 97±3 | 98±3.3 | 45±2 | 69±12 |
| COR L23 | 94±2 | 94±5 | 12±6 | 25±18 |
| MCF-7 | 89±1 | 95±4 | 60±4 | 89±12 |
| T47D | 94±4 | 97±2 | 6±18 | 30±19 |

Thymidine (100 μM) and hypoxanthine (30 μM) transport was measured over 10 s following the incubation of cells ± DP prior to initiation of transport. Data are mean ± standard deviation from three independent experiments.

### DISCUSSION

The studies described in this paper were performed to determine the sensitivity of four solid tumour cell lines to MTA, end-product reversal of growth inhibition by thymidine and hypoxanthine, and inhibition of end-product reversal by DP. To allow direct comparison of the cell lines, the exposure period for each individual cell line was adjusted so that untreated cells underwent at least three cell doublings. The four cell lines used in this study varied ≥ 20-fold in their sensitivity to MTA, A549 cells ($IC_{50}$ = 640 nM) being the least sensitive followed by MCF7 and T47D cells and COR L23 ($IC_{50}$ = 28 nM) the most sensitive. The $IC_{50}$ data calculated for A549, COR L23 and MCF7 cells, based on semi-logarithmic dilutions of MTA accurately predicted the concentration of MTA to inhibit cell growth inhibition by 50% (Figure 1), whereas that calculated from exposure to logarithmic dilutions of MTA in T47D cells apparently underestimated sensitivity and the calculated $IC_{50}$ inhibited growth by approximately 85% (Figure 1). Previous studies with lometrexol and LY309887 (two GARFT inhibitors) indicate a very similar rank order of sensitivity, i.e. T47D (most sensitive) > CORL23 > MCF7 > A549 cells (least sensitive) (Marshman et al, 1998). Thus, the inter-cell variation in sensitivity to MTA, lometrexol and LY309887 could be due to common factors such as antifolate transport and intracellular polyglutamation.

In order to compare end product reversal data, all cell lines were exposed to equiactive concentrations of MTA, i.e. at the $IC_{50}$ or 10 × the $IC_{50}$ concentration. End product reversal experiments

 *© 2000 Cancer Research Campaign*

revealed that 1 μM thymidine alone completely reversed growth inhibition produced by $IC_{50}$ concentrations of MTA in all four cell lines. At 10 × the $IC_{50}$ concentration cell growth was still inhibited by approximately 50% even when the concentration of thymidine was increased to 10 μM. However, complete rescue from 10 × $IC_{50}$ concentrations of MTA in all four cell lines was achieved by the combination of 1 μM thymidine plus 30 μM hypoxanthine. This result indicates that all four cell lines had functional thymidine and hypoxanthine uptake and salvage pathways, and that both nucleotide precursors are required to overcome the inhibition of de novo nucleotide biosynthesis by MTA. Similar observations have been reported in CCRF-CEM leukaemia, GC3/C1 colon carcinoma and HCT-8 iloecaecal carcinoma cells (Shih et al, 1997). Thus, at $IC_{50}$ concentrations of MTA TS appears to be the principal locus of action whilst at higher MTA concentrations a pre-formed purine is required for complete reversal of growth inhibition suggesting that inhibition of de novo purine synthesis also occurs. This is most likely due to the relative potencies of MTA for folate-requiring enzymes; the inhibitory potency of the pentaglutamate of MTA is 50× and 5× greater for TS (Ki = 1.3 nM) than GARFT (Ki = 65 nM) and DHFR (Ki = 7.2 nM) respectively (Shih et al, 1997). Although not tested in the cell lines used here raltitrexed, which has a 90 000× and 66× greater potency for TS than GARFT and DHFR (Ward et al, 1992; Shih et al, 1997), requires only thymidine for complete reversal of growth inhibition even at high concentrations in various cell types (Jackman et al, 1991; Shih et al, 1997).

The concentrations of thymidine and hypoxanthine used in this study are slightly higher than those found in normal human plasma. In cancer patients thymidine levels have been shown to range between 0.07 and 0.8 μM (Howell et al, 1981; Taylor et al, 1984; Traut, 1994) and hypoxanthine levels have been recorded at 0.9 μM (Wung and Howell, 1984). However, significant variations in purines and pyrimidines are found in different tissues (Traut, 1994) and, in contrast to the in vitro setting, there is a continual supply of these salvageable components. Furthermore, hypoxanthine and thymidine concentrations in tumours may be greater than in plasma due to release from dead and dying cells. Thus, tumour cells are likely to be continuously exposed to salvageable nucleosides and bases, and nucleoside and base salvage therefore has the potential to reduce the efficacy of MTA in cancer patients.

Dipyridamole has previously been shown to prevent thymidine and/or hypoxanthine reversal of methotrexate cytotoxicity in a variety of cell types (Nelson and Drake, 1984; Van Mouwerik et al, 1987; Hughes and Tattersall, 1989; Chan and Howell, 1990). In order to determine if inhibition of hypoxanthine uptake by DP has any bearing on the prevention of reversal in MTA-treated cells, the effect of DP on thymidine and hypoxanthine rescue was tested in the four cell lines which have previously been characterized as having either DP sensitive (A549 and MCF7) or DP insensitive (COR L23 and T47D) hypoxanthine rescue from antipurine antifolate-induced growth inhibition (Marshman et al, 1998). DP not only inhibited the partial rescue of MTA-induced growth inhibition by thymidine alone, but also the complete rescue produced by the combination of thymidine and hypoxanthine in all cell lines tested. As hypoxanthine alone did not reverse MTA-induced growth inhibition the inhibition of thymidine salvage alone is sufficient to explain the prevention of thymidine and hypoxanthine rescue by DP.

The combination of MTA + DP, in the presence or absence of thymidine and hypoxanthine, was significantly more growth inhibitory than MTA alone in A549 cells. This enhancement of MTA activity is probably due to DP-mediated inhibition of deoxyuridine efflux as previously observed in A549 cells exposed to the antifolate TS inhibitor CB3717 (Curtin and Harris, 1988). Subsequently, it was shown that in CB3717-treated A549 cells inhibition of deoxyuridine efflux by DP lead to an increased intracellular dUTP pool, DNA strand break levels and cell death (Curtin et al, 1991).

To investigate the mechanism underlying the inhibition of thymidine and hypoxanthine rescue by DP, the effect of DP on the uptake of thymidine and hypoxanthine was determined in all four cell lines. Hypoxanthine transport was not significantly inhibited by 1 μM DP in T47D or COR L23 cells; however, transport was inhibited by 45% and 60% in A549 and MCF7 cells, respectively, consistent with the reported sensitivity (A549 and MCF7) and insensitivity (COR L23 and T47D) to DP of hypoxanthine-mediated reversal from LY309887 growth inhibition (Marshman et al, 1998).

The data presented here indicate that DP can inhibit the reversal of MTA-induced growth inhibition by thymidine and hypoxanthine in both non-small-cell lung cancer and breast cancer cell lines, irrespective of the dipyridamole-sensitivity of their hypoxanthine transport. Inhibition of nucleoside and base transport by DP may therefore represent a novel means of enhancing the anti-tumour activity of MTA. However, attempts to use DP clinically to potentiate antimetabolite activity have so far proved unsuccessful (Wadler et al, 1987; Wilson et al, 1989). A significant problem with the clinical use of DP is the avid binding of DP to the serum protein $\alpha_1$ acid glycoprotein (Mahoney et al, 1982). There is a wide variation in $\alpha_1$ acid glycoprotein in humans, with normal levels between 0.5 and 1 mg $ml^{-1}$ and levels in cancer patients generally being elevated to 1–3 mg $ml^{-1}$ (Kremer et al, 1988). In vitro, potentiation of the growth inhibitory activity of CB3717 by DP was reduced in a concentration-dependent manner by $\alpha_1$ acid glycoprotein (Curtin et al, 1989), and the high $\alpha_1$ acid glycoprotein levels in cancer patients may therefore explain the disappointing clinical results obtained so far with DP. DP analogues that lack $\alpha_1$ acid glycoprotein binding are currently being developed and these compounds may allow the clinical evaluation of MTA given in combination with a nucleoside and base transport inhibitor.

### ACKNOWLEDGEMENTS

This work was funded by Eli Lilly and Company and supported by the Cancer Research Campaign.

### REFERENCES

Calvert AH and Walling JM (1998) Clinical studies with MTA. *Br J Cancer* **78:** 35–40

Chan TCK and Howell SB (1990) Role of hypoxanthine and thymidine in determining methotrexate plus dipyridamole cytotoxicity. *Eur J Cancer* **26**: 907–911

Chen TR (1977) In situ detection of mycoplasma contamination in cell cultures by fluorescent Hoechst 33258 stain. *Exp Cell Res* **104**: 255–262

Curtin NJ and Harris AL (1988) Potentiation of quinazoline antifolate (CB3717) toxicity by dipyridamole in human lung carcinoma, A549, cells. *Biochem Pharmacol* **37**: 2213–2120

Curtin NJ, Newell DR and Harris AL (1989) Modulation of dipyridamole action by $\alpha_1$ acid glycoprotein: reduced potentiation of quinazoline antifolate (CB3717) cytotoxicity by dipyridamole. *Biochem Pharmacol* **38**: 3281–3288

© 2000 Cancer Research Campaign 

Curtin NJ, Harris AL and Aherne GW (1991) Mechanism of cell death following thymidylate synthase inhibition: 2′-deoxyuridine-5′-triphosphate accumulation, DNA damage, and growth inhibition following exposure to CB3717 and dipyridamole. *Cancer Res* **51**: 2346–2352

Howell SB, Mansfield SJ and Taetle R (1981) Significance of variation in serum thymidine concentration for the marrow toxicity of methotrexate. *Cancer Chemother Pharmacol* **5**: 221–226

Hughes JM and Tattersall MHN (1989) Potentiation of methotrexate lymphocytotoxicity in vitro by inhibitors of nucleoside transport. *Br J Cancer* **59**: 381–384

Jackman AL, Taylor GA, Gibson W, Kimbell R, Brown M, Calvert AH, Judson IR and Hughes LR (1991) ICI D1694, a quinazoline antifolate thymidylate synthase inhibitor that is a potent inhibitor of L1210 tumour cell growth in vitro and in vivo: a new agent for clinical study. *Cancer Res* **51**: 5579–5586

Kinsella AR and Haran MS (1991) Decreasing sensitivity to cytotoxic agents parallels increasing tumorigenicity in human fibroblasts. *Cancer Res* **51**: 1855–1859

Kremer JMH, Wilting J and Janssen LHM (1988) Drug binding to human alpha-1-acid glycoprotein in health and disease. *Pharmacol Rev* **40**: 1–47

Mahoney C, Wolfram KM, Cocetto DM and Bjornsson TD (1982) Dipyridamole kinetics. *Clin Pharmacol Ther* **31**: 330–338

Marshman E, Newell DR, Calvert AH, Dickinson AM, Patel HRH, Campbell FC and Curtin NJ (1998) Dipyridamole potentiates antipurine antifolate activity in the presence of hypoxanthine in tumour cells but not in normal tissues in vitro. *Clin Cancer Res* **11**: 2895–2902

Nelson JA and Drake S (1984) Potentiation of methotrexate toxicity by dipyridamole. *Cancer Res* **44**: 2493–2496

Paterson ARP, Kolassa N and Cass CE (1981) Transport of nucleoside drugs in animal cells. *Pharmacol Ther* **12**: 515–536

Plagemann PGW and Wohlhueter RM (1984*a*) Hypoxanthine transport in mammalian cells: cell type-specific differences in sensitivity to inhibition by dipyridamole and uridine. *J Membrane Biol* **81**: 255–262

Plagemann PGW and Wohlhueter RM (1984*b*) Nucleoside transport in cultured mammalian cells: multiple forms with different sensitivity to inhibition by nitrobenzylthioinosine or hypoxanthine. *Biochim Biophys Acta* **773**: 39–52

Shih C, Chen VJ, Gossett LS, Gates SB, MacKeller WC, Habeck LL, Shackelford KA, Mendelsohn LG, Soose DJ, Patel VF, Andis SL, Bewley JR, Rayl EA, Moroson BA, Beardsley GP, Kohler W, Ratnam M and Schultz RM (1997) LY231514, a pyrrolo[2,3-d]pyrimidine-based antifolate that inhibits multiple folate-requiring enzymes. *Cancer Res* **57**: 1116–1123

Taylor GA, Jackman AL, Calvert AH and Harrap KR (1984) Plasma nucleoside and base levels following treatment with the new thymidylate synthase inhibitor CB3717. *Purine Metabolism in Man IV* **165B**: 379–382

Traut TW (1994) Physiological concentrations of purines and pyrimidines. *Mol Cell Biochem* **140**: 1–22

Turner RN, Aherne GW and Curtin NJ (1997) Selective potentiation of lomotrexol growth inhibition by dipyridamole through cell-specific inhibition of hypoxanthine salvage. *Br J Cancer* **76**: 1300–1307

Van Mouwerik TJ, Pangallo CA, Willson JKV and Fischer PH (1987) Augmentation of methotrexate cytotoxicity in human colon cancer cells achieved through inhibition of thymidine salvage by dipyridamole. *Biochem Pharmacol* **36**: 809–814

Wadler S, Subar M, Green MD, Wiernik PH and Muggia FM (1987) Phase II trial of oral methotrexate and dipyridamole in colorectal carcinoma. *Cancer Treat Rep* **71**: 821–824

Ward WHJ, Kimbell R and Jackman AL (1992) Kinetic characteristics of ICI D1694: a quinazoline antifolate which inhibits thymidylate synthase. *Biochem Pharmacol* **43**: 2029–2031

Weber G (1983) Biochemical strategy of cancer cells and the design of chemotherapy: GHA Clowes Memorial Lecture. *Cancer Res* **43**: 3466–3492

Wilson JKV, Fischer PH, Remick SC, Tutsch KD, Grem JL, Nieting L, Alberti D, Bruggink J and Trump DL (1989) Methotrexate and dipyridamole combination chemotherapy based upon inhibition of nucleoside salvage in humans. *Cancer Res* **49**: 1866–1870

Wolhueter RM, Graff JC and Plagemann PGW (1978) A rapid-mixing technique to measure transport in suspended animal cells: applications to nucleoside transport in Novikoff rat hepatoma cells. *Methods Cell Biol* **20**: 211–236

Wung WE and Howell SB (1984) Hypoxanthine concentrations in normal subjects and patients with solid tumours and leukaemia. *Cancer Res* **44**: 3144–3148

*© 2000 Cancer Research Campaign*
